# Photosynthetic performance and growth responses of *Liriope muscari* (Decne.) L.H. Bailey (*Asparagaceae*) planted within poplar forests having different canopy densities

**DOI:** 10.1186/s12898-020-00294-7

**Published:** 2020-04-30

**Authors:** J. J. Zhang, L. Zhu, X. Zhang, J. Zhou

**Affiliations:** 1 College of Biology and the Environment, Nanjing Forestry University, Nanjing, 210037 China; 2 Co-Innovation Center for Sustainable Forestry in Southern China, Forestry University, Nanjing, 210037 China

**Keywords:** Undergrowth, Light acclimation, Light response curve, Chlorophyll fluorescence, Biomass, Correlation analysis

## Abstract

**Background:**

*Liriope muscari* (Decne.) L.H. Bailey is a valuable horticultural and medicinal plant that grows under a range of light intensities, from high to low, in the understories of shrubs. To understand how this species adapts to these various environments, we selected two groups of lilyturf growing under poplar trees at two different spacings. Each group was divided into three types, open field, forest edge and shaded forest with high, medium and low irradiance levels, respectively, and then we examined their photosynthetic characteristics, physiology and biomasses.

**Results:**

Light saturation point, light compensation point and in situ net photosynthetic rate (*P*_N_) were highest in lilyturf growing under high light. In contrast, lilyturf growing under low light had a higher apparent quantum yield and Chl a and b contents, indicating that they adapted to low light. Although the leaves of lilyturf growing under low light were small, their root tubers were heavier.

**Conclusions:**

The research demonstrates the eco-physiological basis of lilyturf’s shade adaptation mechanism as indicated by photosynthetic activity, chlorophyll fluorescence, Chl a, Chl b and Car contents when grown under different irradiances. We believe that lilyturf is a shade-tolerant plant suitable for planting in undergrowth, but attention should be paid to the canopy density of the forest when interplanting. The findings presented here advance our understanding of the photosynthetic characteristics of understory plants and may assist in the optimization of irradiances in the future.

## Background

With the increasing shortage of land resources, it is necessary to make full use of forest resources, attach importance to three-dimensional planting and vigorously develop the undergrowth economy, which improves the utilization rate of land and also promotes tree growth through the management of the undergrowth. In addition, understory plants provide important ecosystem services by supporting tree-seedling regeneration, soil nutrient cycling [1–4] and non-wood forest products (e.g., *Vaccinium myrtillus*) for harvest and recreational enjoyment [5].

Light is a critical resource for plants, particularly in forest understory environments where long periods of low intensity diffuse light are interspersed by brief high intensity light flecks [6]. Acclimation to a light environment is important because photosynthesis is closely related to the production of dry mass [7, 8]. Therefore, understanding plant responses to light has been a long-term focus of plant eco-physiological research [9]. Compared with trees, herbaceous plants are more sensitive to environmental changes. Chai found that *Camellia nitidissima* is a shade-adapted plant with poor adaptability to high light environments [10]. Under-forest illumination conditions are key factors that limit the growth of herbaceous plants in small-scale environments [11]. The distribution of herbaceous plants and their responses to illumination can reveal herbaceous plants’ ecological demands for light and also provide key references for their introduction and utilization. Most understory plants have numerous shade-adaptations, such as epidermal chloroplasts [12], low light compensation points [13], relatively thin, horizontally oriented leaves [14, 15], high leaf longevity [16, 17] and small size at reproductive maturity, allowing a low relative biomass cost of light interception [17, 18]. In the past, research on shade tolerance mainly focused on crops and flowers, with limited reports on the shade tolerance of undergrowth plants in the wild.

*Liriope muscari* (Decne.) L.H. Bailey (*Asparagaceae*), commonly known as big blue lilyturf (or simply “lilyturf”), is a widespread perennial herb native to forests of boreal and temperate East Asia, including parts of China, Korea, and Japan [19]. It is a common undergrowth plant. This species typically grows 30–45-cm tall and has grass-like evergreen foliage. Because it is easily grown and has broad tolerance for a wide range of soil, light, temperature and moisture conditions [20, 21], lilyturf is widely used for horticultural purposes, particularly as a border plant or groundcover. It also has a strong resistance to environmental pollution [22, 23]. Consequently, lilyturf has been planted well beyond its natural range and has become naturalized in parts of southeastern North America and elsewhere. At present, The Global Biodiversity Information Facility (https://www.gbif.org/) lists 1885 records for *L. muscari* (and synonyms) worldwide. Most of these records are from warm and humid areas that are often forested. Lilyturf likes a sparsely shaded environment and grows well in the shade, making it suitable for planting in the lower layer of multi-layer configurations of trees, shrubs and flowers. Thus, it is an ideal ground cover plant to create a natural ecology by effectively covering the bare soil under the tree. Consequently, the landscape space under the forest is embellished to improve the ecological benefit.

There is limited literature addressing the ecophysiology of lilyturf, except some works on seed germination [24]. Because the roots and tubers of lilyturf are used for a variety of medicinal purposes, most studies on this species have focused on its biological evolution [25, 26], chemical composition [27–29], pharmacology and pharmacodynamics [30–32]. Although lilyturf can grow under either high light in the open field or low illumination associated with the understory, little is known about the leaf photosynthetic characteristics and responses to irradiance.

In our study, lilyturf growing under poplar trees was the research subject used to analyze the responses of this species to the illumination factor in the forest. Our goals were to determine the mechanism whereby this plant acclimates to changes in irradiance and evaluate the relative importance of leaf physiology to their photosynthetic adaptations, to provide a theoretical basis for the development and cultivation of undergrowth plants.

## Results

### Environmental conditions

**Fig. 1:**
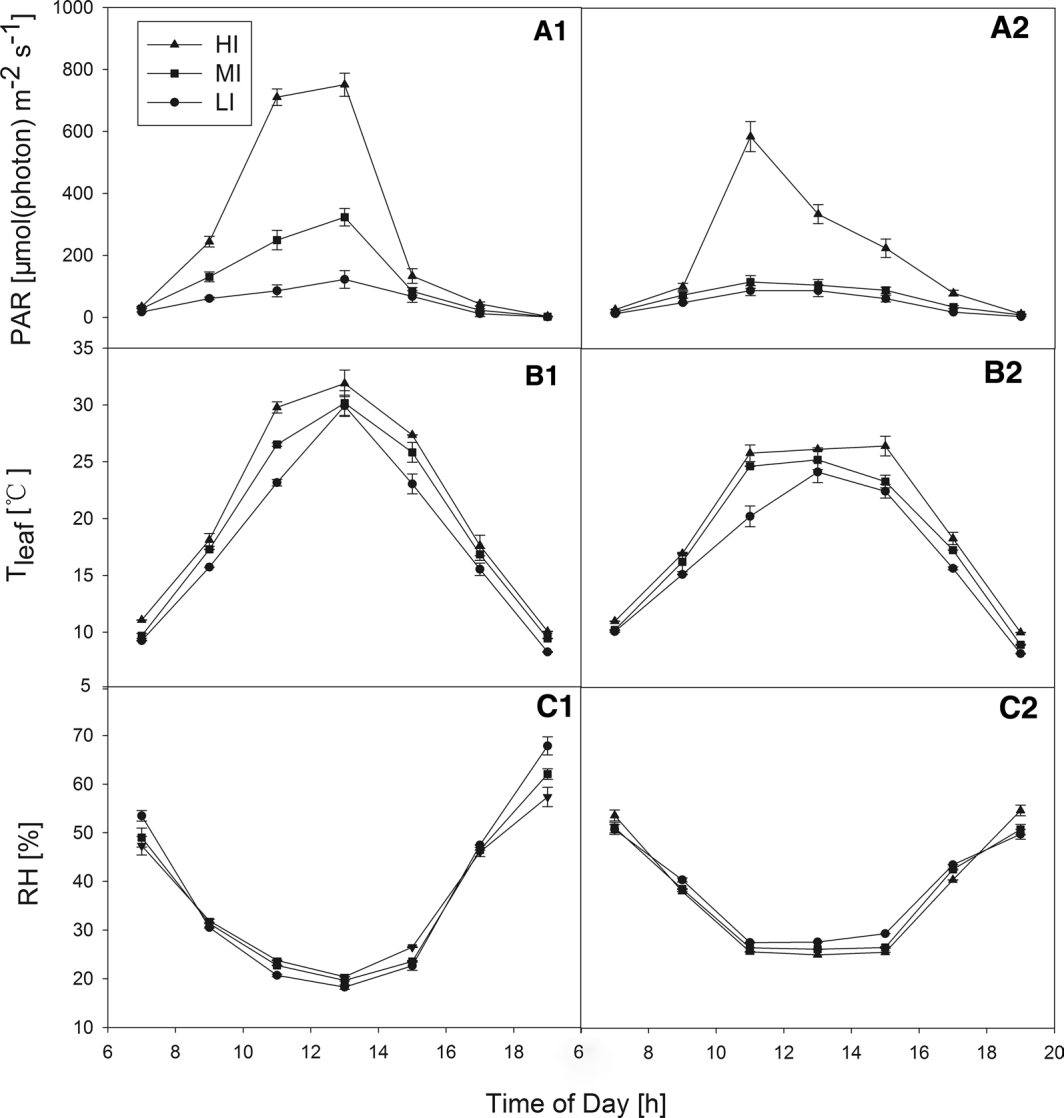
Diurnal changes in **A1-2** photosynthetically active radiation (PAR), **B1-2** leaf temperature (T_leaf_), and **C1-2** relative humidity (RH) inside the cuvette under different shade treatments in group1 (**A1**, **B1**, **C1**) and group2 (**A2**, **B2**, **C2**). Mean ± SE (n = 3)

PAR followed a normal daily course. The maximum illumination value occurred at 13:00 h for all the treatments in the first group, while for the second group it occurred at 11:00 h (Fig. 1A1-2). The highest PAR observed inside the cuvette was 751 µmol (photon) m^−2^ s^−1^ at 13:00 h under HI conditions in first group. The three PAR values of the first group were all greater than those in the second group, and the difference in PAR between MI and LI plants in the second group was not significant.

Patterns in leaf temperature (T_leaf_) followed a similar trajectory. The maximum T_leaf_ ranged from 30 to 32 °C in the first group and 24**–**26 °C in the second group, depending on the light environment (Fig. 1B1-2). T_leaf_ declined in the middle to late afternoon. In the same group, the T_leaf_ of HI plants was greater than those of MI and LI plants, but the difference was not significant. Under the same light conditions, the T_leaf_ of the first group was slightly higher than that of the second group, which was related to the PAR of the first group being slightly greater than that of the second group.

Under all the conditions, the trends in relative humidity (RH) were opposite those of temperature, with the lowest values recorded at 13:00 h (Fig. 1C1-2). The RH of the first group was slightly lower than that of the second group during midday, while the RH levels under HI, MI and LI conditions in the same group were not difference.

### Light-response curves under different growth irradiances

**Fig. 2:**
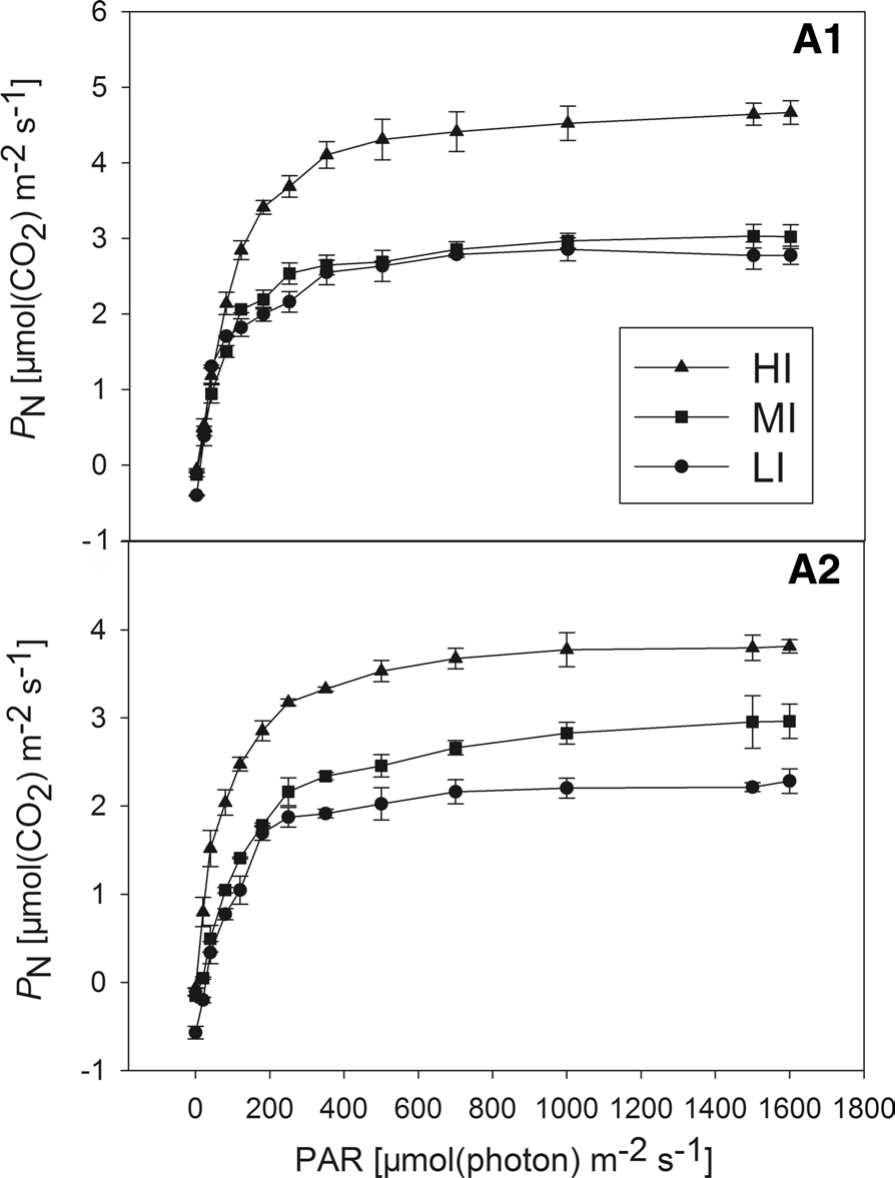
Response of net photosynthetic rate (*P*_N_) to PAR in *Liriope muscari* grown under high (HI), medium (MI) and low (LI) incident PAR in group1 (**A1**) and group2 (**A2**). Mean ± SE (n = 3)

The dark respiration rate of HI-treated plants measured at zero light was greater than those of MI- and LI-treated plants (p < 0.05) in the same group (Table 1). There were clear differences in light responses as PAR increased. In the first group, the light-response curve began to level off faster in the LI- and MI-treated plants than in the HI-treated plants, and the *P*_N_ rates of HI-treated plants were significantly greater than those of MI- and LI-treated plants (Fig. 2A1). In the second group, from 0 to 200 µmol (photon) m^−2^ s^−1^, all the curves responded rapidly, and then, increased slowly to their maximum values (Fig. 2A2). Moreover, P_N_ rates of HI-, MI- and LI-treated plants decreased successively. In all the treatments, the net CO_2_ assimilation increased as the light intensity decreased until the PPFD was 700 µmol (photon) m^−2^ s^−1^. In same group, *P*_Nmax_, LSP and LCP increased as growth irradiance increased. The AQY was highest in the LI-treated plants of the second group and lowest in the HI-treated plants of first group, but these differences were not statistically significant (Table 1).

**Table 1:** Comparison of photosynthetic characteristics (Pmax—photon-saturated photosynthetic rate; AQY—apparent quantum yield; LSP—light saturation point; LCP—light compensation point; Rd—dark respiration rate) of Liriope muscari grown under high (HI), medium (MI) and low (LI) incident PAR in two groups

Traits	1			2		
	HI	MI	LI	HI	MI	LI
Pmax [µmol m^**−**2^ s^**−**1^]	4.81 ± 0.09^a^	3.33 ± 0.02^b^	3.09 ± 0.06^c^	4.05 ± 0.08^ab^	3.14 ± 0.09^b^	2.93 ± 0.03^c^
AQY [mol mol^**−**1^]	0.031 ± 0.003^b^	0.041 ± 0.004^b^	0.047 ± 0.003^ab^	0.038 ± 0.006^a^	0.043 ± 0.01^ab^	0.053 ± 0.001^ab^
LSP [µmol m^**−**2^ s^**−**1^]	492.80 ± 50.29a	441.60 ± 5.54ab	400.00 ± 22.40abc^c^	480.00 ± 19.20ab	398.67 ± 8.73^c^	363.73 ± 11.88^bc^
LCP [µmol m^**−**2^ s^**−**1^]	11.73 ± 0.56^a^	6.51 ± 0.01^c^	3.20 ± 0.01^c^	6.40 ± 0.01^b^	4.30 ± 0.01^c^	2.73 ± 0.56^a^
Rd [µmol m^**−**2^ s^**−**1^]	0.569 ± 0.04^d^	0.151 ± 0.03b	0.065 ± 0.02^ab^	0.393 ± 0.01^c^	0.120 ± 0.02^ab^	0.060 ± 0.01^a^

Mean ± SE (n = 3). Different superscripted letters following the values in each column indicate significant differences (p < 0.05) among the different growth irradiances

### Photosynthesis and carbon economy

**Fig. 3:**
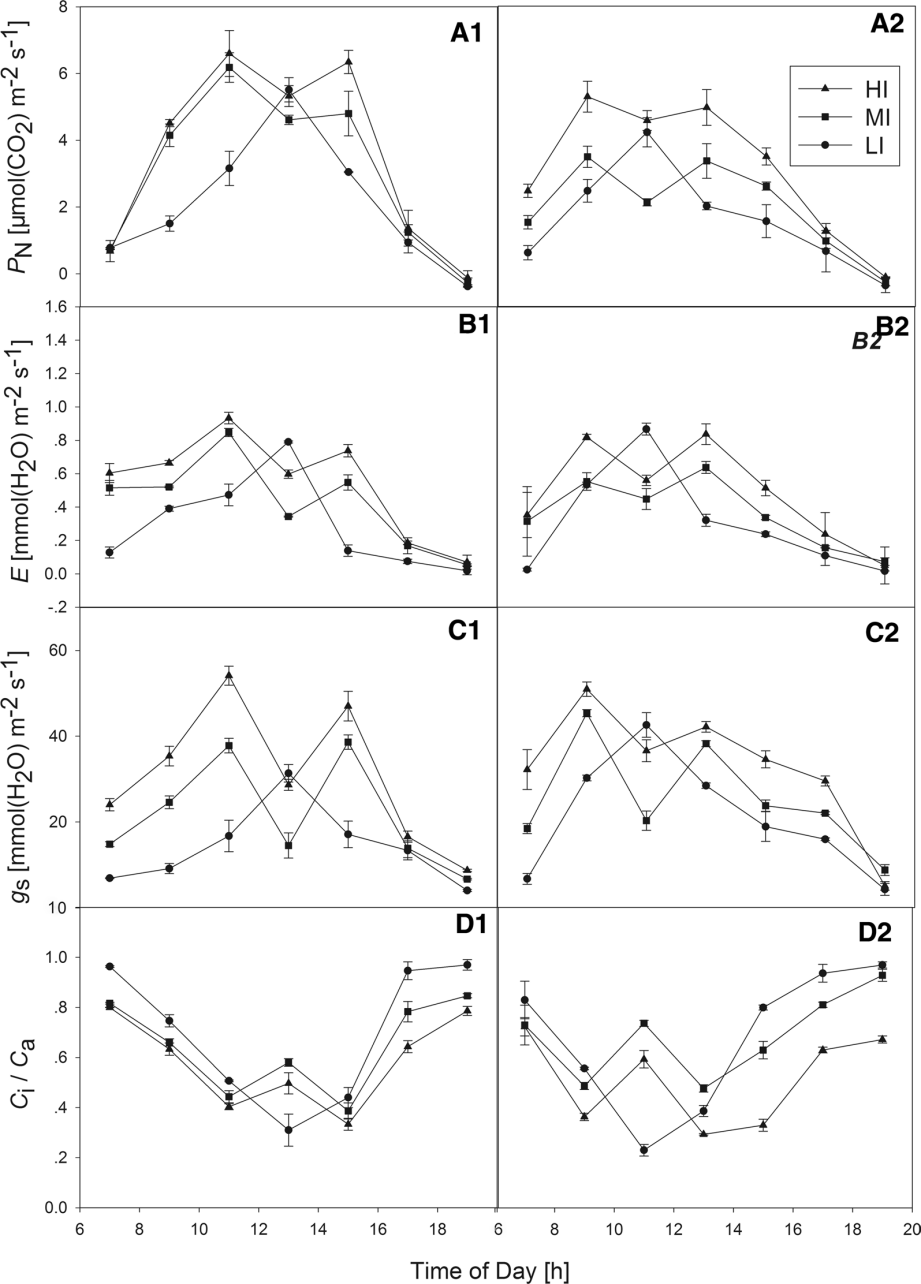
Changes in **A1-2** net photosynthetic rate (*P*_N_), **B1-2** transpiration rate (*E*), **C1-2** stomatal conductance (*g*_s_), and **D1-2** internal to ambient CO_2_ concentration (*C*_i_/*C*_a_) of *Liriope muscari* plants grown under high (HI), medium (MI) and low (LI) incident PAR in group1 (**A1**, **B1**, **C1**, **D1**) and group2 (**A2**, **B2**, **C2**, **D2**). Mean ± SE (n = 3)

Under HI and MI conditions, lilyturf showed a typical two-peak pattern of diurnal changes in photosynthesis (Fig. 3A). In the first group, the first peak was in mid-morning (~ 11:00 h), while the second smaller peak was in the middle to late afternoon (~ 15:00 h). In the second group, there was a midday depression, and the two peaks occurred about 2 h earlier (~ 9:00 h and 13:00 h). However, under LI conditions, the diurnal variation of *P*_N_ was a single-peak curve, and the peak value occurred just as the midday depression found under HI and MI conditions emerged. During daylight, the *P*_N_ in HI-treated plants was significantly higher than in MI-treated plants, and that of LI-treated plants was always significantly lower than in the other two treatments, except during their midday depressions. When comparing the two groups, the *P*_N_ value of the first was slightly higher than that of the second. Although some light was still available, the PAR was below compensation at 19:00 h, resulting in similar marginally negative *P*_N_ values under all the treatment conditions.

The change trend of *E* was also the same as that of *P*_N_ in both groups. Overall, *E* was low in the morning (Fig. 3B), increased towards midday and declined in the late afternoon. Similarly, the *E* value of HI-treated plants was higher than those of MI- and LI-treated plants during the day, and there was no significant difference in the *E* values between the two groups.

The diurnal *g*_*s*_ patterns were similar to those of *P*_N_ in all the treatments, and by the evening, the *g*_s_ values were lower than the starting values in the morning (Fig. 3C). In the same group, the *g*_*s*_ value under HI conditions was also higher than under MI and LI conditions, except during the midday depression. There were no large differences in *g*_s_ between the two groups. As a whole, the *g*_s_ values of HI-treated plants in the first group were larger and fluctuated greatly, with the highest value appearing at 11:00 h.

The *C*_i_ to *C*_a_ showed opposite trends to the *P*_N_. *C*_i_/*C*_a_ values were higher in the morning and evening for all the treatments, which may in part reflect lower T_leaf_ values and higher cuvette RH levels. No significant differences in *C*_i_/*C*_a_ were observed among all the irradiance conditions in the first group. However, in the second group, the differences in *C*_i_/*C*_a_ among the three kinds of light were more obvious. HI-treated plants had lower values than MI- and LI-treated plants, except at 11:00 h, and they ranged from ~ 0.29 to 0.45 during the early afternoon (Fig. 3D2).

To investigate the cause and effect relationship, we performed a path analysis of *P*_N_ with each factor. The maximum decision coefficient is the main determinant, and the negative and the minimum coefficients are the main limiting factors. The direct path coefficient of environmental factors on *P*_N_ in HI- and MI-treated plants of the first group was as follows: RH > T_leaf_ > PAR, and the maximum decision coefficient was RH, while in LI-treated plants, the maximum decision coefficient was T_leaf_. In the second group, the direct path coefficient of environmental factors on *P*_N_ and the maximum decision coefficient in MI- and LI-treated plants were both PAR, while in HI-treated plants, they were both T_leaf_ and RH was the major limiting factor (Table 2). In the path analysis of physiological factors’ effects on *P*_N_, the maximum direct path coefficient and decision coefficient in HI- and MI-treated plants of the first group were both *C*_i_/*C*_a_, while for LI-treated plants, they were both *g*_s_. In the second group, the direct path coefficient of physiological factors on *P*_N_ and the maximum decision coefficient in HI- and LI-treated plants were both *E*, with *g*_s_ being the major limiting factor (Table 3).

**Table 2:** Path coefficients between Pn and environmental factors of *Liriope muscari* grown under high (HI), medium (MI) and low (LI) incident PAR in two groups

Treatment	Variable	Direct effect	Indirect effect				Decision coefficient
			PAR X1	Tleaf X2	RH X3	∑	
HI-1	PAR X1	− 0.09	–	0.128	0.684	0.812	− 0.651
	Tleaf X2	0.154	− 0.075	–	0.830	0.755	− 0.547
	RH X3	− 0.888	0.069	− 0.144	–	− 0.075	0.783
Regression equation: Y = 7.617 + 0.001 * X1 + 0.049 * X2 − 0.131 * X3							
MI-1	PAR X1	0.032	–	− 0.090	0.851	0.760	− 0.577
	Tleaf X2	− 0.108	0.027	–	0.967	0.993	− 0.975
	RH X3	− 1.035	− 0.026	0.101	–	0.075	1.066
Regression equation: Y = 9.317 + 0.001 * X1 − 0.032 * X2 − 0.157 * X3							
LI-1	PAR X1	0.383	–	0.562	–	0.562	− 0.170
	Tleaf X2	0.608	0.354	–	–	0.354	0.244
Regression equation: Y = − 1.519 + 0.017 * X1 + 0.152 * X2							
HI-2	Tleaf X2	− 3.630	–	–	4.287	4.287	0.657
	RH X3	− 4.326	–	3.597	–	3.597	− 0.729
Regression equation: Y = 48.844 − 1.044 * X2 − 0.682 * X3							
MI-2	PAR X1	1.4	–	− 0.263	− 0.371	− 0.634	1.558
	Tleaf X2	− 0.279	1.320	–	− 0.377	0.943	− 0.812
	RH X3	0.384	− 1.354	0.274	–	− 1.080	− 1.019
Regression equation: Y = 0.262 + 0.07 * X1 − 0.094 * X2							
LI-2	PAR X1	1.293	–	− 0.314	− 0.141	− 0.456	1.464
	Tleaf X2	− 0.363	1.120	–	− 0.140	0.980	− 0.828
	RH X3	0.147	− 1.243	0.346	–	− 0.897	− 0.783
Regression equation: Y = − 0.337 + 0.056 * X1 − 0.091 * X2 + 0.022 * X3

**Table 3:** Path coefficients between Pn and physiological factors of Liriope muscari grown under high (HI), medium (MI) and low (LI) incident PAR in two groups

Treatment	Variable	Direct effect	Indirect effect				Decision coefficient
			E X1	GS X2	ci/ca X3	∑	
HI-1	E X1	0.391	–	–	0.434	0.434	− 0.036
	ci/ca X3	− 0.665	− 0.255	–	–	− 0.255	0.377
Regression equation: Y = 7.603 + 3.583 * X1 − 10.276 * X3							
MI-1	E X1	0.418	–	− 0.339	0.683	0.343	0.057
	GS X2	− 0.408	0.348	–	0.879	1.226	− 1.338
	ci/ca X3	− 0.985	− 0.290	0.364	–	0.074	0.965
Regression equation: Y = 11.581 + 3.854 * X1-0.08 * X2 − 13.086 * X3							
LI-1	E X1	0.161	–	0.369	0.325	0.693	− 0.455
	GS X2	0.462	0.128	–	0.365	0.493	− 0.030
	ci/ca X3	− 0.426	− 0.123	-0.395	–	− 0.518	− 0.087
Regression equation: Y = 2.453 + 1.149 * X1 + 0.1 * X2 − 3.029 * X3							
HI-2	E X1	1.062	–	0.021	− 0.122	− 0.101	1.118
	GS X2	0.024	0.938	–	− 0.101	0.837	− 0.699
	ci/ca X3	0.154	− 0.844	− 0.016	–	− 0.860	− 0.716
Regression equation: Y = − 1.427 + 7.388 * X1 + 0.003 * X2 + 1.726 * X3							
MI-2	GS X2	0.507	–	–	0.385	0.385	0.109
	ci/ca X3	− 0.457	–	− 0.427	–	− 0.427	0.026
Regression equation: Y = 2.961 + 0.055 * X2 − 3.454 * X3							
LI-2	E X1	0.545	–	0.412	–	0.412	0.127
	GS X2	0.434	0.518	–	–	0.518	− 0.080
Regression equation: Y = − 0.224 + 2.677 * X1 + 0.048 * X2							

### Chl fluorescence

**Fig. 4:**
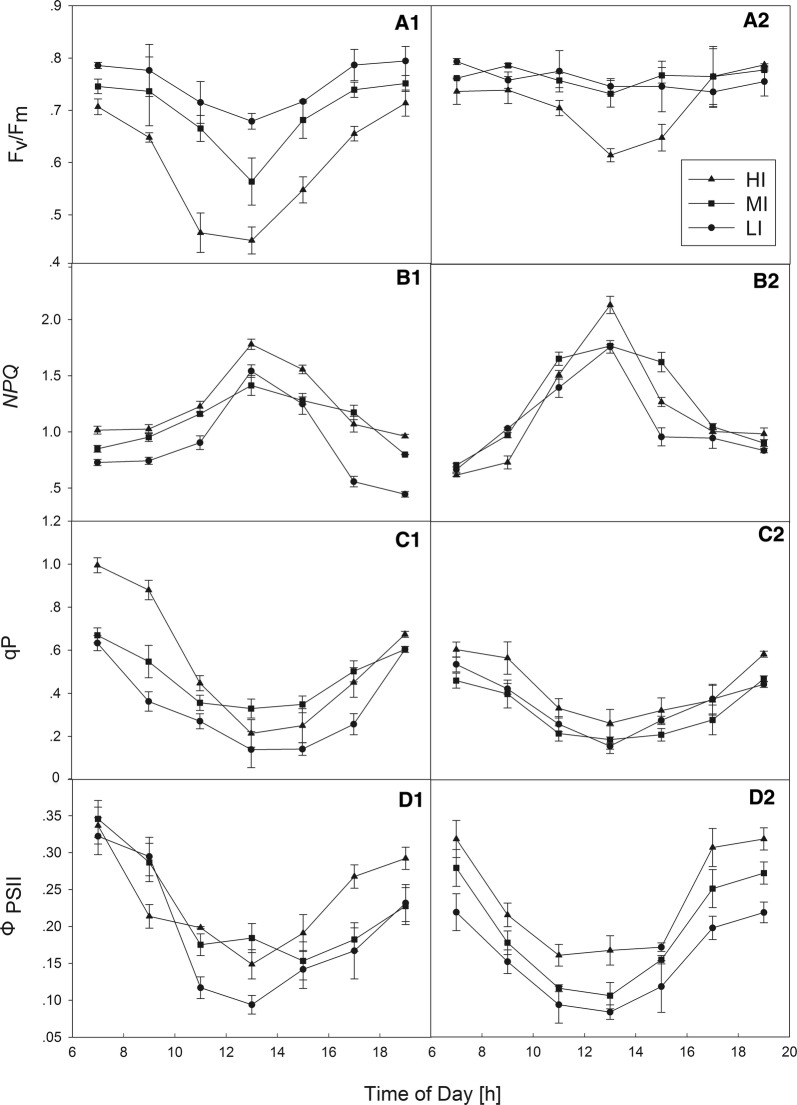
Diurnal changes in **A1-2** maximal photochemical efficiency (F_v_/F_m_), **B1-2** nonphotochemical quenching (NPQ), **C1-2** photochemical quenching (qP), and **D1-2** quantum yield of PSII (Ф_PSII_) of *Liriope muscari* plants grown under high (HI), medium (MI) and low (LI) incident PAR in group1 (**A1**, **B1**, **C1**, **D1**) and group2 (**A2**, **B2**, **C2**, **D2**). Mean ± SE (n = 3)

In the first group, calculated fluorescence parameters under LI conditions were more stable over the course of the day than under MI and, particularly, HI conditions. The F_v_/F_m_ under LI conditions remained relatively high (> 0.67) from dawn until dusk. In contrast, there was a decrease in F_v_/F_m_ observed under MI and HI conditions, which reached minimum values of 0.56 and 0.45, respectively, at 13:00, followed by a recovery in the late afternoon to values near 0.75 after sunset (Fig. 4A1). In the second group, under LI and MI conditions, F_v_/F_m_ remained stable, ranging from 0.75 to 0.78, and there was no significant difference between values under the two conditions. Under HI conditions, F_v_/F_m_ decreased significantly during the midday period (Fig. 4A2). In general, the fluctuation range in the F_v_/F_m_ values of the second group was smaller than that of the first group.

Regardless of the treatments, the q_p_ value was lower from the late morning until midday than in the late afternoon (Fig. 4C1,2). In the first group, the q_p_ in LI-treated plants was lower than under the other two treatment conditions during the day, while in the second group, there were no significant differences. There was a larger increase in NPQ at midday owing to the decrease in q_p_ at this time (Fig. 4B1,2). The NPQ in HI-treated plants was significantly higher during midday (13:00) than in the morning or afternoon. In the second group, treatments were similar at dusk and at dawn.

Concurrently, in the two groups, the Ф_PSII_ in all the treatments dropped significantly at 13:00 pm, and recovered during the late afternoon. Otherwise, the Ф_PSII_ value of second group was highest under HI conditions and lowest under LI conditions (Fig. 4D2).

### Chl content

In the same group, LI-treated plants had significantly greater Chl a, Chl b and Chl (a + b) contents than HI- and MI-treated plants. When comparing the two groups, the Chl content in the second group was significantly higher than in the first group (Table 4). The highest value of Chl (a + b) was found in LI-treated plants of second group, and this value was three times higher than the lowest value (HI-treated plants of first group). The Car content was consistent with the Chl a and Chl b contents. LI-treated plants had higher levels than MI- and LI-treated plants, but there was no significant difference between the two groups. In contrast, the higher the degree of shading, the lower the Chl a/b value.

**Table 4:** Chl content, biomass and leaf properties (mean ± SE, n = 3) of Liriope muscari grown under high (HI), medium (MI) and low (LI) incident PAR in two groups

Traits	1			2		
	HI	MI	LI	HI	MI	LI
Chl a [mg L^−1^]	4.71 ± 0.78b	5.30 ± 0.21ab	11.77 ± 2.93ab	6.69 ± 0.24ab	8.57 ± 0.52ab	13.59 ± 3.06a
Chl b [mg L^−1^]	1.60 ± 0.20c	2.08 ± 0.15c	5.15 ± 1.07b	2.44 ± 0.07c	3.43 ± 0.12bc	7.68 ± 0.64a
Chl (a + b) [mg L^−1^]	6.31 ± 0.57b	7.38 ± 0.25b	16.92 ± 3.98ab	9.14 ± 0.25b	12.01 ± 0.61ab	21.26 ± 3.70a
Chl a/b	2.91 ± 0.14a	2.56 ± 0.20a	2.25 ± 0.10a	2.74 ± 0.13a	2.50 ± 0.10a	1.73 ± 0.24b
Caro [mg L^−1^]	2.05 ± 0.29a	2.46 ± 0.37a	3.52 ± 0.16a	2.47 ± 0.07a	2.86 ± 0.06a	3.77 ± 0.81a
whole plant weight [g]	6.84 ± 1.53a	6.48 ± 1.36a	6.89 ± 0.22a	6.08 ± 0.80a	5.78 ± 1.24a	6.65 ± 1.02a
Root weight [g]	3.11 ± 0.84a	3.34 ± 0.79a	3.20 ± 0.12a	3.35 ± 0.46a	2.91 ± 0.67a	3.57 ± 1.06a
Root tubers weight [g]	0.32 ± 0.13ab	0.34 ± 0.08ab	0.88 ± 0.45a	0.19 ± 0.01b	0.25 ± 0.03b	0.43 ± 0.10ab
Root tubers length [cm]	1.73 ± 0.19ab	2.20 ± 0.10ab	2.53 ± 0.52a	1.30 ± 0.15b	1.73 ± 0.24ab	2.00 ± 0.06ab
Leaf weight [g]	0.14 ± 0.03a	0.14 ± 0.04a	0.14 ± 0.03a	0.19 ± 0.01a	0.12 ± 0.01a	0.18 ± 0.01a
Long leaf length [cm]	34.47 ± 0.64a	32.60 ± 0.49a	34.67 ± 0.68a	34.20 ± 0.40a	31.33 ± 0.58a	32.97 ± 1.48a
Long leaf width [cm]	0.60 ± 0.15a	0.57 ± 0.15a	0.40 ± 0.01a	0.43 ± 0.03a	0.43 ± 0.03a	0.33 ± 0.03a
Short leaf length [cm]	21.20 ± 0.72a	17.67 ± 2.08ab	17.83 ± 0.20ab	18.27 ± 1.33ab	17.43 ± 0.55ab	15.47 ± 0.45b
Short leaf width [cm]	0.37 ± 0.03a	0.40 ± 0.10a	0.33 ± 0.03a	0.43 ± 0.10a	0.40 ± 0.03a	0.27 ± 0.03a

Different letters in superscript following the values in each row indicate significant differences (p < 0.05)

### Growth and biomass

By separating the lilyturf, the weights of whole plants, roots, leaves and root tubers were obtained (Table 4). There was no significant difference in the whole-plant weight of the same group, and the weight of the second group was slightly less than that of the first group. The differences in root and leaf weights were also not obvious, accounting for 45–55% and 2–3% of the whole-plant weight, respectively. The HI-treated plants in the second group had the highest percentage of root to whole-plant weight and leaf to whole-plant weight, at 55.1% and 3.1%, respectively.

In the same group, the average root tuber weight of LI-treated plants was higher than that of HI- and MI-treated plants. The average weight of root tubers in the first group accounted for ~ 10% of the root weight, while the difference in the second group ranged from 6 to 24%. LI-treated plants in the second group had the highest root tubers weight, accounting for 24.6% of the root weight. Root tuber lengths and weights showed basically the same trends. LI-treated plants had longer tubers than HI- and MI-treated plants, and those of the LI-treated plants of the second group were the longest, at 2.53 cm (Table 4).

We measured the lengths and widths of the longest and shortest leaves in each plant. There was no significant difference in the longest leaf lengths among the six treatments. The longest leaf width of the first group was slightly greater than that of the second group. The HI-treated plants in the first group had the longest short-leaf length, but a difference in short-leaf width was not obvious.

### Soil characteristics

By measuring the soil around the lilyturf under different light conditions, we found that the N and K contents in the surrounding soil were significantly different under different conditions (Table 5). The N and K contents in the nearby soil of the first group were significantly greater than those of the second group. In each group, the N and K contents in soils near LI-treated plants were greater than those in soils near MI- and HI-treated plants. The greatest N and K values appeared in the soils near LI-treated plants in the first group, and the lowest values appeared in the soils near HI-treated plants in the second group. The former were 5 and 16 times higher, respectively, than the latter.

**Table 5:** N, K, organic matter content and pH of the soil around *Liriope muscari* grown under high (HI), medium (MI) and low (LI) incident PAR in two groups (mean ± SE, n = 3)

Traits	1			2		
	HI	MI	LI	HI	MI	LI
N [mg kg^−1^]	9.50 ± 0.06d	29.83 ± 0.96b	43.03 ± 0.97a	8.60 ± 0.06d	9.50 ± 0.03b	12.83 ± 0.03c
K [mg kg^−1^]	9.20 ± 0.64b	15.00 ± 1.35b	41.03 ± 1.36a	2.47 ± 0.62d	6.83 ± 0.71cd	15.50 ± 1.10b
Organic matter content [%]	0.37 ± 0.03c	0.63 ± 0.03ab	0.77 ± 0.03a	0.23 ± 0.03c	0.37 ± 0.03c	0.57 ± 0.03b
pH	7.58 ± 0.08a	7.37 ± 0.11ab	7.58 ± 0.07a	7.25 ± 0.02b	7.04 ± 0.01b	7.27 ± 0.01b

Different letters in superscript following the values in each row indicate significant differences (p < 0.05)

The soil organic matter content was consistent with the variations in N and K contents. The content was higher in the first group than the second group, and the HI-treated plants in each group had greater soil organic matter contents than the MI- and LI-treated plants. The highest value was 3.3 times the lowest value.

However, the acid-alkalinity of these soils were not very different, being basically neutral and slightly alkaline, and ranging from pH 7.04 to 7.58 (Table 5).

## Discussion

Lilyturf shows a classic pattern of acclimation to a wide range of light environments. Based on the regression of the maximum *P*_N_ and the relatively low saturating irradiance, we confirmed that lilyturf is a shade-tolerant species.

Shade reduces the production of ATP in the PSII reaction center by inhibiting the electron flow rate, resulting in a decrease in photosynthetic rate [33]. Plants grown at low irradiance for a long time may have lower contents of electron transfer components and photosynthetic enzymes in comparison with plants grown at high irradiance, which causes the *P*_Nmax_ decrease [34]. LSP and LCP are the important traits for photon energy utilization capability, and their declines are thought to allow adaptation to low irradiance conditions. Here, we found that the *P*_Nmax_, LSP and LCP of HI-treated plants were all greater than in MI- and LI-treated plants, and the values of the first group were higher than those of the second group, which was consistent with the irradiance environments. The AQY can reflect the ability of leaves to use low irradiance [35]. The AQY of LI-treated plants was greater, indicating that there may be more pigment protein complexes absorbing and converting light energy and that their ability to utilize low irradiance is stronger. Decreased Rd is beneficial to maintain carbon balance in plants under low light conditions in which photosynthetic rates are limiting [36]. Here, we determined that LI-treated plants had the lower Rd values than MI- and HI-treated plants.

Lilyturf experienced a pronounced depression in *P*_N_ under HI and MI conditions at midday, unlike under LI conditions (Fig. 3A), and the midday depression of both groups appeared with the highest illumination and temperature of the day, at 13:00 and 11:00 h, respectively. This phenomenon has been reported in many species [37–39]. An increase in *C*_i_/*C*_a_ in response to changes in *P*_N_ and a decrease in *g*_s_ indicates the strong contribution of non-stomatal limitation to carbon photosynthetic uptake [40]. As shown in Fig. 3, our data showed a decrease in *g*_s_ that was associated with reductions in *P*_N_ and *E* and an increase in *C*_i_/*C*_a_ at midday. Thus, the decrease in *P*_N_ under HI and MI conditions at midday was not caused by stomatal limitation, and *g*_s_ should not limit photosynthesis in these conditions. The high irradiance (well above saturation) or the associated increase in temperature may have caused the decrease in *C*_i_ in the HI- and MI-treated plants. In addition, *P*_N_ and *g*_s_ increased, while *C*_i_/*C*_a_ decreased in both groups’ HI- and MI-treated plants at midday, consistent with an increased biochemical limitation in photosynthesis. In addition, we speculate that photorespiration may also have some effect on the net photosynthetic rate at midday during high light illumination. Photorespiration is the major electron sink for the dissipation of excess excitation energy [41]. The coordination of photorespiration and cyclic electron flow is important for sustaining leaf photosynthesis [42]. In the second group, the difference in *P*_N_ between HI- and MI-treated plants was greater than in the first group, which may be related to the larger difference in illumination between HI and MI conditions in the second group.

Path coefficients between *P*_N_ and environmental factors showed that the main decision factor for the *P*_N_ of MI- and LI-treated plants in the second group was PAR, which was related to the low-irradiance environments of the two groups. In the first group, in which HI- and MI-treated plants had sufficient light, RH was the main influencing factor, while light and temperature were the limiting factors. Through a path analysis of the physiological factors’ influences on *P*_N_, it was determined that the main decision factor in HI- and MI-treated plants in the first group growing under strong illumination was *C*_i_/*C*_a_, while *g*_s_ was the most decisive factor when illumination was weak, such as in LI-treated plants in the first group and MI-treated plants in the second group.

The Fv/Fm ratio indicates the intrinsic efficiency of PSII photochemistry. A reduction in Fv/Fm is often taken to indicate photoinhibition [43]. In the first group, Fv/Fm decreased at 13:00 h under all three conditions, and the decline was greater when the illumination was stronger. However, in the second group, Fv/Fm decreased significantly at midday only in HI-treated plants (Fig. 4A). Under shading conditions, the Fv/Fm ratios remained constant (~ 0.78), at a value that was slightly lower than the average value found in leaves of a wide range of C3 species (0.83; [44]). Thus, shading might protect the integration of the photosynthetic membrane systems and photochemical efficiency in leaves of lilyturf against the strong light stress during midday, and in an open environment, the combination of a high PPFD level and temperature during midday might damage the PSII apparatus in the leaves of this species.

Non-photochemical quenching reflects the ability of plants to dissipate excess light energy into heat and is involved in regulating and protecting photosynthesis in environments in which the amount of absorbed light energy exceeds the utilization capacity [45]. Under all conditions, lilyturf had a higher NPQ at midday, coincident with the depression in q_p_. In both groups, the NPQ of HI-treated plants was significantly higher than those of MI- and LI-treated plants at 13:00 h. Thus, under strong light, the amount of light energy absorbed by the photosynthetic apparatus of plants exceeds the amount used by photosynthesis, leading to a decrease in the proportion of light energy absorbed for photochemical reactions [46]. The photosynthetic activity of the PSII reaction center decreased, therefore, the excess light energy dissipated in the form of heat energy, forming a light protection mechanism to avoid the damage of excessive light to plant photosynthetic organs.

Photosynthetic pigments play an important role in absorbing and transferring light energy during photosynthesis. Under a shade environment, plants can increase photon absorption and compensate for the lower radiant energy by increasing the pigment content per unit area [37]. In this study, the Chl a, Chl b and Car contents of lilyturf under LI conditions were greater than under MI and HI conditions, and those in the second group were higher than those in the first group, indicating that the increase in photosynthetic pigment content was helpful in the plants’ adaptation to a low-light environment. The significant increases in Chl a, Chl b and Chl (a + b) contents in LI-treated plant’ leaves compared with those of HI- and MI-treated plants most likely resulted from changes in both photon harvesting and electron transport components [47]. The increase in the Chl b content can also absorb the short-wavelength blue-violet light in the diffuse light, thus helping plants to capture more light energy for photosynthesis and growth [48].

In previous studies, we found that plants usually have smaller PSII antenna sizes under high-light conditions, while under low-light conditions plants have larger PSII antenna sizes [49]. This is because the amounts of the outer PSII antenna proteins (the major peripheral antenna proteins) change in response to light conditions, while the inner PSII antenna proteins (the core and inner peripheral antenna proteins) remain unchanged [50]. Thus, we found that the Chl a/b ratio is lower in LI-treated plants, as has been documented in many studies, as in the sun and shade leaves of forest trees [51].

Resources other than light might also limit the performance of plants in forest understory environments [52]. Just as the amount of nutrients in the soil affects plant growth, the growth of plant roots also affects the soil. The root systems of plants are the source of soil organic matter, which provides the environment for the activities of soil microorganisms and has a great influence on soil texture, structure, water conditions and nutrient contents.

The N, K and organic matter contents in the soil near LI-treated plants were the highest, which may be related to LI-treated plants growing close to the trunks of poplar trees. The roots of the tree fix the soil nutrients. In addition, the N, K and organic matter contents in the soil of the first group were higher than those of the second group, which was related to the density of poplar trees in the latter.

The morphological characteristics of lilyturf’ biomass accumulation and distribution were significantly affected by shading stress. Root tubers growing under LI conditions were the heaviest, accounting for 24.6% of the root weight, and the root tuber lengths were longer, which might be related to more nutrients in the soil near LI-treated plants. Additionally, under shading conditions, greater dry matter accumulation in the root tubers enhances the supply of plant nutrients and making them more adaptable to the limited light conditions [53]. However, in terms of leaf lengths and widths, leaves of HI-treated plants were longer and wider than those of MI- and LI-treated plants, and the first group had slightly higher values than the second group, indicating that light played a role in promoting leaf growth.

## Conclusion

In this study, although HI-treated plants had higher *P*_N_ values in diurnal variation of photosynthesis (Fig. 3), the Chl content and light-response parameters showed that lilyturf grown under LI conditions was more adaptable to low light (Tables 1 and 4). This suggested that lilyturf is a shade-tolerant species. Although phenotypic plasticity tends to be low in LI-treated plants (e.g., leaf sizes in low light), plasticity for certain traits, such as morphological features optimizing light capture, can be high in these plants. In addition, we found the weights and lengths of the root tubers of the LI-treated plants were greater than those of the HI-treated plants. This is also related to the lilyturf growing under LI conditions, which are near the roots of poplars, where the soil nutrients were relatively high (Table 5). When planted with poplar, the denser poplar resulted in less light and soil nutrients, which impacted the photosynthesis and biomass of lilyturf. Therefore, for the plants like lilyturf with higher plasticity, we can use its shade tolerance and adopt a form of interplanting with poplars or other trees to carry out extensive understory planting, resulting in the maximum benefit. The findings presented here advance our understanding of the photosynthetic characteristics of lilyturf and may assist in the optimization of irradiances needed to improve its productivity in the future.

## Methods

### Plants and experimental design

Lilyturf plants were raised from sprouted tubers planted during July 2013 into an adjacent open field, forest edge and shaded forest understory on the Chenwei Forest Farm in Sihong County (33°15′ N, 118°18′ E).

All experimental plants were cultivated in the early years by our research group and obtained the experimental permit from Chenwei Forest Farm.

Lilyturf plants growing under poplar with two different row spacings were measured. The first group of lilyturf grown under poplar trees had a spacing of 8 m, the open field plants received a maximum of approximately 751 µmol (photon) m^−2^ s^−1^ of photosynthetically active radiation (PAR) on clear days, and the forest edge and shaded forest plants received approximately 44% and 19% of the incident PAR, respectively. The second group of lilyturf grown under poplar had a row spacing of 4 m, the open field plants received a maximum of approximately 585 µmol (photon) m^−2^ s^−1^ of PAR, and the forest edge and shaded forest plants received approximately 33% and 24% of the incident PAR, respectively, not including sun flecks. The light environments (treatments) of the open field, forest edge and forest understory, were designated as high irradiance (HI), medium irradiance (MI) and low irradiance (LI), respectively. Plants were watered regularly during the summer months, but irrigation was not required after the onset of the monsoon rains. All the observations were taken after 5 years on clear days in autumn (October 28–31, 2018). Measurements were made on three well-separated (0.5–1 m) plants per treatment (18 plants in total).

### Gas-exchange measurements

Photosynthesis was measured under natural light, temperature, and humidity conditions on clear days (October 28 and 29, 2018) at 2-h intervals from 7:00 to 19:00 h [i.e., from after sunrise (6:39 h) to after sunset (17:19 h)]. The net photosynthetic rate (*P*_N_), stomatal conductance (*g*_s_), intercellular CO_2_ concentration (*C*_i_), ambient chamber CO_2_ concentration (*C*_a_) and transpiration rate (*E*) were measured using a hand-held GFS-3000 photosynthesis system (Heinz Walz GMBH, Effeltrich, Germany). The central green portion of leaves was always used for measurements.

### Light-response curves

The response of *P*_N_ to step changes in PAR was examined using a red + blue LED light source (GFS-3000, Heinz Walz GMBH). The light-response curve measurements were carried out on the mornings of October 30 and 31, 2018. The *C*_a_ and air temperature in the leaf chamber were maintained at ~ 450 µmol (CO_2_) mol^−1^ and 15 °C, respectively. Thirteen irradiances [0, 20, 40, 80, 120, 180, 250, 350, 500, 700, 1000, 1400 and 1500 µmol (photon) m^−2^ s^−1^ PAR] were used, beginning at 0 µmol (photon) m^−2^ s^−1^ and increased stepwise to 1500 µmol (photon) m^−2^ s^−1^. Leaves were allowed to acclimate to each PAR for at least 3 min, and then, steady-state gas-exchange properties were observed and recorded. Light-response curves were plotted using the mean values of *P*_N_ measured at each PAR. There were three replicates per treatment, using one leaf from each of the plants measured for time courses the previous day. The apparent quantum yield (AQY) was calculated from the initial slopes by linear regression using PAR values below 200 µmol (photon) m^−2^ s^−1^. The light compensation point (LCP), light saturation point (LSP), dark respiration rate (Rd) and *P*_Nmax_ were estimated using the methods of Bassman and Zwier [54].

### Chlorophyll (Chl) fluorescence

After at least 20 min of dark adaptation, the minimal level of Chl a fluorescence (F_0_) was measured using a Junior-PAM fluorometer (Heinz Walz GmbH) under a modulated light intensity of 0.1 µmol (photon) m^−2^ s^−1^. The maximum level of Chl a fluorescence (F_m_) was induced by applying a 0.6 s flash of saturating light [10,000 µmol (photon) m^−2^ s^−1^]. This was followed by actinic PAR of 190 µmol (photon) m^−2^ s^−1^ with pulses of saturating light applied at 20 s intervals for 4 min. Variable fluorescence (F_v_) was calculated as F_m_ **−** F_0_, and the maximum quantum yield of Photosystem (PS) II (F_v_/F_m_) was obtained accordingly [55]. The quantum yield of PSII electron transport was determined using Ф_PSII_ = (${\text{F}}_{\text{m}}^{{\prime }}$ − F_s_)/${\text{F}}_{\text{m}}^{{\prime }}$. Nonphotochemical quenching (NPQ) was calculated as F_m_/${\text{F}}_{\text{m}}^{{\prime }}$ − 1. Photochemical quenching (q_p_) was calculated as (${\text{F}}_{\text{m}}^{{\prime }}$ − F_s_)/(F_m_ − ${\text{F}}_{0}^{{\prime }}$) according to Schreiber et al. [56].

### Biomasses, leaf properties and Chl contents

Three plants per treatment were harvested for the determination of the whole plant fresh biomass using an electronic scale. Then, the whole plant was divided into three parts: leaves, roots and root tubers, and independently weighed. The lengths of root tubers were also measured. Immediately after harvesting, the three longest and shortest leaves of each plant were selected to determine the lengths and widths.

To determine the Chl concentration, fresh leaf samples of 100 mg were taken from fully expanded leaves of each experimental unit (container). The samples were immediately transferred to a freezer and kept at − 30 °C for subsequent measurements. The samples were ground and extracted with a 10-mL mixture of 4.5: 4.5:1 ethanol:acetone:water in the dark for 24 h. Determinations of Chl a, Chl b and carotenoid (Car) concentrations were performed at 645,663 and 470 nm, respectively, using a spectrophotometer (U-2802 UV/VIS, Unico, China) with 80% acetone as the blank. Concentrations of Chl a, Chl b, and Car were determined and expressed as mg L^−1^ leaf fresh weight in accordance with Lichtenthaler and Wellburn [57].

### Soil characteristics

Soil samples around lilyturf grown under nine conditions were collected, and for each condition, three soil samples were collected. The 0–20-cm depth of each soil sample was air-dried naturally and screened. Then, 5 g of sifted soil sample was placed into a soil sample bottle, and 20 mL of water and 0.5 g of the determination powder were added. The bottle was shaken for 1 min and then stood for 2 min. It was shaken again and filtered. The resulting liquid was measured. Distilled water, distilled water containing 2 drops of a nitrogen (N) [potassium (K)] standard liquid, or organic matter standard liquid, and soil liquid were placed in three separate cuvettes, representing the zero standard liquid, calibration liquid and liquid to be tested, respectively. Then, the soil N content, soil K content and soil organic matter content were measured using a soil nutrient tester (SL-3C-1 multifunctional, Shunlong, China). Three repeats were conducted per sample.

Then, 10 g of screened soil was placed in a beaker, 25 mL of water was added, and it was stirred evenly. Then, the solution was allowed to rest for 30 min. The pH value of the soil was measured using the pH electrode of the soil tester. Three repeats were conducted per sample.

### Data analyses

All the data are presented as means ± SEs. Differences in measured variables between treatments in time were analyzed by two-factor ANOVA using SigmaPlot software 12.0 (Systat Software Inc., San Jose, CA, USA), and the means were compared using Student–Newman–Keuls multiple comparison tests. All the tests for significance were performed at p < 0.05, unless otherwise indicated.

Correlation coefficients between *P*_N_ and each factor were calculated by correlation analyses, and data distribution normality was determined before the analyses. Then, a path analysis of *P*_N_ with each factor was carried out to calculate the direct and indirect effects of different factors on *P*_N_. The main influencing factors were determined by calculating the decision coefficients. A decision coefficient is a decision index in the path analysis, and it is used to sort the comprehensive effects of the corresponding variables of each factor, and finally determine the main determinants and limiting factors [58]. The calculation formula is as follows:

$${{\text{R}}^2}\left( {\text{I}} \right) = 2{{\text{P}}_{{\text{iy}}}}{{\text{r}}_{{\text{iy}}}} - {\text{P}}_{{\text{iy}}}^2,$$

where R^2^(I) represents the decision coefficient of the variable y for the argument x, P_iy_ represents the direct path coefficient, and P_iy_r_iy_ represents the indirect path coefficient. All the data were obtained using Excel 2016 (Microsoft, Redmond, WA, USA) and SPSS17.0 software [IBM (R) Corporation, Somers, NY, USA] for processing.

## Data Availability

The datasets of this study will be available from corresponding authors on genuine request.

## Abbreviations

AQY: Apparent quantum yield
*C*_a_: Ambient chamber CO_2_ concentration
*C*_i_: Intercellular CO_2_ concentration
CCI: Chlorophyll content index
*E*: Transpiration rate
F_0_: Minimal fluorescence yield of the dark-adapted state
F_m_: Maximal fluorescence yield of the dark-adapted state
${\text{F}}_{\text{m}}^{{\prime }}$: Maximal fluorescence yield of the light-adapted state
F_s_: Steady-state fluorescence yield
F_v_: Variable fluorescence
F_v_/F_m_: Maximum photochemical efficiency of PSII
*g*_s_: Stomatal conductance
LCP: Light compensation point
LSP: Light saturation point
NPQ: Nonphotochemical quenching
*P*_Nmax_: Light-saturated photosynthetic rate
*P*_N_: Net photosynthetic rate
PS: Photosystem
qP: Photochemical quenching coefficient
Rd: Dark respiration rate
Ф_PSII_: Actual photochemical efficiency of PSII
